# Prevalence and correlates of treatment failure among Kenyan children hospitalised with severe community-acquired pneumonia: a prospective study of the clinical effectiveness of WHO pneumonia case management guidelines

**DOI:** 10.1111/tmi.12368

**Published:** 2014-08-14

**Authors:** Ambrose Agweyu, Minnie Kibore, Lina Digolo, Caroline Kosgei, Virginia Maina, Samson Mugane, Sarah Muma, John Wachira, Mary Waiyego, Elizabeth Maleche-Obimbo

**Affiliations:** 1 Department of Paediatrics and Child Health, University of NairobiNairobi, Kenya; 2 Kenya Medical Research Institute – Wellcome Trust Research ProgrammeNairobi, Kenya

**Keywords:** treatment failure, case management, World Health Organization, pneumonia

## Abstract

**Objective:**

To determine the extent and pattern of treatment failure (TF) among children hospitalised with community-acquired pneumonia at a large tertiary hospital in Kenya.

**Methods:**

We followed up children aged 2–59 months with WHO-defined severe pneumonia (SP) and very severe pneumonia (VSP) for up to 5 days for TF using two definitions: (i) documentation of pre-defined clinical signs resulting in change of treatment (ii) primary clinician's decision to change treatment with or without documentation of the same pre-defined clinical signs.

**Results:**

We enrolled 385 children. The risk of TF varied between 1.8% (95% CI 0.4–5.1) and 12.4% (95% CI 7.9–18.4) for SP and 21.4% (95% CI 15.9–27) and 39.3% (95% CI 32.5–46.4) for VSP depending on the definition applied. Higher rates were associated with early changes in therapy by clinician in the absence of an obvious clinical rationale. Non-adherence to treatment guidelines was observed for 70/169 (41.4%) and 67/201 (33.3%) of children with SP and VSP, respectively. Among children with SP, adherence to treatment guidelines was associated with the presence of wheeze on initial assessment (*P* = 0.02), while clinician non-adherence to guideline-recommended treatments for VSP tended to occur in children with altered consciousness (*P* < 0.001). Using propensity score matching to account for imbalance in the distribution of baseline clinical characteristics among children with VSP revealed no difference in TF between those treated with the guideline-recommended regimen *vs*. more costly broad-spectrum alternatives [risk difference 0.37 (95% CI −0.84 to 0.51)].

**Conclusion:**

Before revising current pneumonia case management guidelines, standardised definitions of TF and appropriate studies of treatment effectiveness of alternative regimens are required.

**Objectif:**

Déterminer l'ampleur et les caractéristiques de l’échec du traitement (ET) chez les enfants hospitalisés avec une pneumonie acquise dans la communauté dans un grand hôpital tertiaire du Kenya.

**Méthodes:**

Nous avons suivi des enfants âgés de 2 à 59 mois avec une pneumonie sévère (PS) et une pneumonie très sévère (PTS) telles que définies par l’OMS, sur un maximum de cinq jours pour l’ET, en utilisant deux définitions: (a) documentation des signes cliniques prédéfinis ayant entraîné un changement du traitement, (b) décision primaire du clinicien de changer de traitement avec ou sans documentation des mêmes signes cliniques prédéfinis.

**Résultats:**

Nous avons recruté 385 enfants. Le risque d’ET variait de 1,8% (IC95%: 0,4 à 5,1) à 12,4% (IC95%: 7,9 à 18,4) pour la PS et de 21,4% (IC95%: 15,9 à 27) à 39,3% (IC95%: 32,5 à 46,4) pour la PTS selon la définition appliquée. Des taux plus élevés étaient associés à des changements précoces du traitement par le clinicien en l'absence d'une justification clinique évidente. Le non-respect des directives de traitement a été observé pour 70/169 (41,4%) et 67/201 (33,3%) enfants avec une PS et une PTS respectivement. Chez les enfants avec une PS, le respect des directives de traitement était associé avec la présence d'une respiration sifflante au cours l’évaluation initiale (P = 0,02) tandis que le non respect par les cliniciens des traitements recommandés pour la PTS tendait à se produire chez les enfants avec une altération de la conscience (P <0,001). L'utilisation du score de propension correspondant pour tenir compte du déséquilibre dans la répartition des caractéristiques cliniques de base chez les enfants avec une PTS n'a révélé aucune différence dans l’ET entre ceux traités avec le régime recommandé par les directives et ceux traités par des alternatives plus coûteuses à large spectre (différence de risque: 0,37 (IC95%: -0,84 à 0,51).

**Conclusion:**

Avant la révision des directives actuelles de prise en charge des cas de pneumonie, des définitions standard d’ET et des études appropriées de l'efficacité des traitements alternatifs sont nécessaires.

**Objetivo:**

Determinar la extensión y el patrón del fallo en el tratamiento (FT) en niños hospitalizados con una neumonía adquirida en la comunidad, ingresados en un gran hospital terciario de Kenia.

**Métodos:**

Hemos seguido a niños con edades entre los 2-59 meses con una neumonía severa (NS) y neumonía muy severa (NMS) según definición de la OMS de hasta cinco días para FT utilizando dos definiciones: (a) documentación de signos clínicos pre-definidos que resultaron en un cambio de tratamiento (b) decisión del clínico principal de cambiar el tratamiento con o sin documentación de los mismos signos clínicos pre-definidos.

**Resultados:**

Incluimos a 385 niños. El riesgo de FT varió entre un 1.8% (IC 95% 0.4 a 5.1) y 12.4% (IC 95% 7.9 a 18.4) para NS y 21.4% (IC 95% 15.9 a 27) y 39.3% (IC 95% 32.5 a 46.4) para NMS dependiendo de la definición que se aplicase. Unas mayores tasas estaban asociadas con cambios tempranos en la terapia por el clínico y en ausencia de un razonamiento clínico obvio. Se observaba una no adherencia a las guías de tratamiento en 70/169 (41.4%) y 67/201 (33.3%) de los niños con NS y NMS respectivamente. Entre los niños con SP, la adherencia a las guías de tratamiento estaba asociada con la presencia de sibilancias en la evaluación inicial (P=0.02) mientras que la no adherencia del clínico a los tratamientos recomendados por las guías para NMS tendían a ocurrir en niños con un estado alterado de consciencia (P<0.001). Utilizando el pareamiento por puntaje de propensión para equilibrar los grupos en la distribución de las características clínicas de base de los niños con NMS, se observó que no existían diferencias en FT entre aquellos tratados con el régimen recomendado por las guías versus alternativas más costosas de amplio espectro (diferencias de riesgo 0.37 (IC 95% -0.84 a 0.51).

**Conclusión:**

Antes de revisar las actuales guías de manejo de casos de neumonía, se requieren definiciones estandarizadas de FT y estudios apropiados de la efectividad del tratamiento de regímenes alternativos.

## Introduction

Pneumonia is the leading cause of childhood mortality, responsible for nearly one and a half million annual deaths (Liu *et al.* 2012). Severe and very severe presentations are very common causes of admission to Kenyan hospitals (Berkley *et al.* 2005) and routine data from Kenyatta National Hospital (KNH) indicate that pneumonia is present in 30% of hospitalised children, who have a case fatality rate of 6.5% (Irimu *et al.* 2012). One strategy currently in place to tackle the high burden of pneumonia is case management. This involves prompt diagnosis and classification of severity of disease and empiric treatment with recommended antibiotics. Using this approach, hospital admission in Africa is still largely aimed at severe and very severe classifications in accordance with guidelines developed 20 years ago by the World Health Organization (WHO 1990). Over this period, accumulated evidence has demonstrated that use of this case management strategy can reduce pneumonia mortality (Sazawal & Black 2003; Niessen *et al.* 2009). Kenya is among a majority of low-income countries, which have adopted the WHO case management guidelines for the management of pneumonia in children (Table 1).

**Table 1:** Kenyan Ministry of Health (MoH) guidelines for management of children aged 2–59 months with cough and/or difficulty breathing (for a child without stridor, severe malnutrition or signs of meningitis)

Syndrome	Clinical signs	Recommended antibiotic treatment
Non-severe pneumonia	Fast breathing (RR ≥50/min if age 2–11 months; ≥40/min if age 12–59 months) AND without signs of severe or VSP	Outpatient treatment Co-trimoxazole (or amoxicillin if child has HIV and is receiving Co-trimoxazole prophylaxis)
Severe pneumonia	Indrawing AND without signs of VSP	Inpatient treatment Benzyl penicillin/ampicillin monotherapy (if HIV-exposed, treat as VSP)
VSP	Any one of: Cyanosis, grunting (infants), SPO_2_ <90%, inability to drink, altered consciousness	Inpatient treatment Benzyl penicillin/ampicillin and gentamicin (*plus* high dose co-trimoxazole for all HIV-exposed)

VSP, very severe pneumonia.

However, recent concerns have been expressed over the continued effectiveness of these guidelines, such as (i) the emergence of antibiotic resistance, particularly to penicillin (Scott *et al.* 1998; Nyandiko *et al.* 2007): (ii) the possibility of a changing spectrum of bacterial pathogens in the face of widespread coverage with the *Haemophilus influenzae* type b (Hib) vaccine (Murphy *et al.* 1993; Mulholland *et al.* 1997; Wenger 1998; Adegbola *et al.* 2005; Cowgill *et al.* 2006; Watt *et al.* 2009) [86.4% national coverage with three doses in Kenya (Government of Kenya 2009)], recent deployment of the 10-valent pneumococcal conjugate vaccine (Scott & English 2008); and (iii) the influence of HIV. Pneumonia is now the leading cause of hospitalisation in HIV-infected children (Zwi *et al.* 1999; Oniyangi *et al.* 2006; Kourtis *et al.* 2007) and is associated with poorer outcomes, and a wider spectrum of pathogens (Madhi *et al.* 2000; Zar *et al.* 2001; Graham 2003; McNally *et al.* 2007).

Against the backdrop of these theoretical concerns, empiric data on rates of treatment failure (TF) and mortality have helped inform discussion around revision of guidelines, particularly in Asia (Addo-Yobo *et al.* 2004; Asghar *et al.* 2008; Hazir *et al.* 2008). Unfortunately, however, approaches to defining TF vary and reported studies include relatively few African children who, on the background of a higher prevalence of HIV and malnutrition, may manifest an atypical pattern of disease associated with poorer outcomes (Graham 2003; Jeena *et al.* 2006). One recent study among children hospitalised at a rural Kenyan hospital reported TF risks of 20% for very severe pneumonia (VSP) and 12% for severe pneumonia (SP) at 48 h (Webb *et al.* 2012). The lack of evidence on the effectiveness of antibiotic treatments currently in use for the management of childhood pneumonia became apparent at a recent national guideline development meeting in Kenya where the Grading of Recommendations Assessment and Development (GRADE) approach was used to generate recommendations using available evidence (Guyatt *et al.* 2008). During this exercise, evidence relating to clinical questions on childhood pneumonia was frequently assigned lower levels of quality owing to a lack of locally generalisable evidence (Agweyu *et al.* 2012). The scarcity of local data on the effectiveness of antibiotic treatments for childhood pneumonia may also be partly responsible for the widespread practice of non-adherence to national guideline recommendations among clinicians treating acute respiratory infections who have been observed to frequently opt for expensive, broad-spectrum regimens (English *et al.* 2004).

Given the potential limitations of currently recommended case management guidelines and a specific paucity of data from African settings, we aimed to describe the extent and pattern of TF in a population of children admitted to KNH with WHO-defined, community-acquired pneumonia during a period 8 years after the national launch of the Hib vaccine and prior to the introduction of the pneumococcal conjugate vaccine. We further sought to compare the clinical outcomes of children with VSP treated in accordance with the national guidelines *vs*. those who received more aggressive regimens. Such data are needed to inform national policy and will hopefully prompt wider efforts to examine the effectiveness of current guidelines, a much neglected topic.

## Methods

We conducted a short prospective longitudinal survey from June to October 2009 in KNH; a large tertiary hospital located in Nairobi (altitude 1700 m) that receives an average of 900 paediatric admissions per month. Despite being a national hospital, a large proportion of patients present directly from home, bypassing lower levels of care.

Children aged 2–59 months satisfying the WHO case definitions for SP or VSP (Table 1) whose caregivers consented to participation were recruited. Those with suspected or confirmed pulmonary tuberculosis, congestive cardiac failure secondary to congenital cardiac disease, chronic cardiopulmonary symptoms (for >14 days), prior treatment with injectable antibiotics within the 2 weeks preceding or gross neurological disorders (for example, cerebral palsy) were excluded. Children with a wheeze whose signs of respiratory distress subsided after up to three cycles of salbutamol nebulisation 15 min apart were also excluded. Screening was undertaken 24 h a day by nine study doctors – eight paediatric residents (trainee paediatricians) and one medical officer. All the investigators were trained in the Kenya national/WHO paediatric case management protocols as part of a 5-day course on inpatient case management (Vella *et al.* 1992; Irimu *et al.* 2008). Children requiring emergency care were attended immediately, and recruitment and data collection were deferred until after stabilisation.

### Sample size estimation

Using estimates from previous Kenyan studies (Maina 2007, unpublished data; Nokes *et al.* 2009), our initial sample size calculations were based on a projected enrolment of a total of 600 children with SP and VSP at a ratio of 3:1, respectively. Such a sample would yield odds ratios of 2 or more for associations of risk factors with outcomes, assuming a risk factor prevalence of 20%, to be identified for a sample size of 200 or greater. Point estimates for prevalence of TF as high as 30% would be estimated within margins of ±4.2% and 7.3% for the SP and VSP groups, respectively.

### Clinical procedures

A standardised history and physical examination was completed for each enrolled child. Details on prior admissions or care, including a check of any patient-held documentation and history of specific, recent antibiotics received were recorded. Severe acute malnutrition was defined by the presence of visible severe wasting or the presence of oedema of both feet due to kwashiorkor.

Oxygen saturation was determined in all patients using a portable pulse oximeter (Nellcor NPB-40). Children with saturations below 90% after breathing ambient air for a minimum of 3 min were deemed hypoxemic and received supplementary oxygen in accordance with local practice.

Blood samples were collected before administration of antibiotics for bacterial culture (BACTEC 9050 system; Becton Dickinson). Routine, rapid HIV testing was requested for all children, according to the Government of Kenya guidelines for provider-initiated testing and counselling (PITC) (Government of Kenya 2009) and confirmatory HIV-1 DNA PCR performed for any child <18 months old with a positive rapid HIV test (Roche AMPLICOR HIV-1 test version 1.5).

A detailed description of the criteria we used to define TF among children with SP and VSP is shown in Table 2. Although investigators recruiting children initiated management, decisions on management from the time of arrival on the ward and during the stay were made by consultant-led teams of clinicians, independent of the investigators, encouraged to adhere to the national (WHO) childhood pneumonia treatment guidelines. Independent follow-up clinical examinations were performed and data on antibiotic treatment recorded by the investigators at 24 and 48 h while only data on antibiotic treatment were collected daily up to day five. TF at day five was reported cumulatively; thus, children who failed treatment at 48 h and later recovered were included among those who failed treatment at day five. Outcomes at final discharge or death were also recorded.

**Table 2:** Definitions of treatment failure

Pneumonia severity classification	Treatment failure: Criterion 1 (*a priori* definition) – any of the numbered criteria listed Criterion 2 (*post hoc* definition) – any of the numbered criteria listed except (iv) for SP and (v) for VSP
SP	(i) Development of signs of VSP or death at any time (ii) Absence of improvement of all of the following: (a) indrawing (persistence), (b) measured temperature reduction of ≥0.5 °C, (c) respiratory rate (reduction of ≥5 bpm) (iii) Identification of pathogen with *in vitro* resistance to the antibiotics at any time point (iv) Senior clinician's decision to change antibiotic treatment or initiate TB treatment following initial treatment allocation shown in Figure 2
VSP	(i) Observed deteriorating level of consciousness (reduction in AVPU), death or development of respiratory failure resulting in the need for ICU transfer at any time point (ii) Chest X-ray findings indicative of lung abscess, bullae formation or pulmonary TB at any time point (iii) Absence of improvement of all of the following: (a) indrawing (persistence), (b) measured temperature reduction of ≥0.5 °C, (c) respiratory rate (reduction of ≥5 bpm), (d) ability to drink, (e) requirement of supplementary oxygen (iv) Identification of a pathogen on blood culture or from pleural fluid with *in vitro* resistance to the antibiotics at any time point (v) Senior clinician's decision to start the child on second line treatment or TB treatment following initial treatment allocation shown in Figure 2

SP, severe pneumonia; VSP, very severe pneumonia.

### Statistical analysis

Completed questionnaires were double-entered and verified using Epidata Version 3.0 (EpiData Association, Odense, Denmark) and data analysed using Stata Version 11 (StataCorp LP, College Station, TX, USA). The primary aims of analysis were to estimate the prevalence and determine the clinical as well as sociodemographical correlates of prospectively-defined TF and mortality. To address the latter aims, chi-square or Fisher's exact test, one-way analysis of variance or the Kruskal–Wallis test was used as appropriate to explore differences in patient groups or initial associations between risk factors and outcomes. We also fitted logistic regression models to identify independent predictors of TF using the *a priori* definition, a modified *post-hoc* definition (described in the results) and cumulative mortality at day 5 post-recruitment. Variables considered to be associated with the outcomes were used to fit the full model, and using a backward stepwise selection procedure with a pre-defined cut-off *P*-value of 0.2, variables were selected out one at a time until the final model was obtained. Adjusted odds ratios for association were then reported with accompanying 95% confidence intervals.

### Propensity score derivation and matching

In post-hoc analyses, we further sought to compare the effectiveness of the guideline-recommended treatments against alternative regimens frequently used by clinicians among children with VSP. To address this objective, crude comparisons of clinical outcomes among children recruited in the study and treated according to the recommended guidelines against those treated with alternative regimens would be prone to selection bias arising from differences in baseline clinical characteristics, which may influence the prescribing patterns of clinicians. Thus, children who were perceived to have more severe forms of disease were more likely to have been assigned treatments perceived to be more effective by the primary clinician and vice versa. To account for this potential bias, we fitted a multivariable logistic regression model with treatment assigned as the dependent variable and sociodemographical/clinical characteristics at the time of admission as covariates to generate a propensity score for each subject adjusting for baseline clinical characteristics among recruited children. Matching patients in the two treatment groups of interest on the propensity scores generated, we derived an estimate of the average treatment effect among those treated with more aggressive regimens for which we calculated a 95% confidence interval.

## Results

### Diagnosis and classification at initial assessment

**Figure 1:**
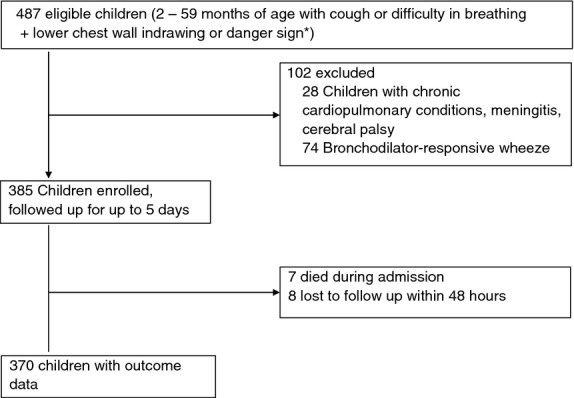
Flow of study patients.

Between June and October 2009, 593 children were admitted at KNH with a diagnosis of probable SP or VSP of whom 487 (82%) were screened for eligibility (Figure 1). Exclusion criteria were identified in 102 (74 had bronchodilator-responsive wheeze and 28 met other exclusion criteria).

**Figure 2:**
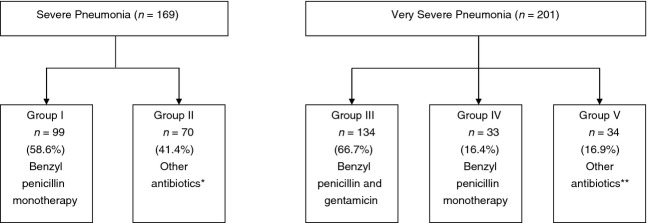
Diagnosis showing subgroups of initial treatment assigned. *Other antibiotics: 53 benzyl penicillin and gentamicin, eight benzyl penicillin and chloramphenicol, two ceftriaxone, three ceftriaxone and amikacin, one cloxacillin and gentamicin, one amoxicillin/clavulanate, one benzyl penicillin, gentamicin and erythromycin, one benzyl penicillin and erythromycin. **Other antibiotics: 18 benzyl penicillin and chloramphenicol, three ceftriaxone, nine ceftriaxone and amikacin, one ceftazidime and amikacin, one ceftazidime, one cloxacillin and erythromycin, one amoxicillin/clavulanate.

Of the 385 enrolled, 171 (44.4%) has SP and 214 (55.6%) VSP. Seven children died during the process of admission before full assessment, and data collection could be completed and were therefore excluded from analyses for TF and mortality. A further eight children (two with SP and six with VSP) were lost to follow-up within 24 h of recruitment. Although the investigating team initiated first-line antibiotic treatment as per the WHO/national guidelines, treatment was changed by ward-based clinical teams (upon the arrival of patients to the wards) and deviated from guidelines in 37.0% (137/370) of cases. The impact of such decisions on overall observed rates of TF is discussed below. Further, this non-adherence to recommended regimens necessitated division of patients into five groups for analysis (Figure 2), rather than the intended two. Baseline characteristics of these children are given in Table 3.

**Table 3:** Baseline sociodemographical and clinical characteristics of recruited children with severe forms of pneumonia

Patient characteristics	All pneumonia frequency (%) or median (IQR)	Total number of patients with characteristic recorded
Female	195 (52.7%)	370
Age in months	8.7 (5.2–15.5)	354
Age 2–11 months	233 (65.8%)	354
12–59 months	121 (34.2%)	191
Duration of illness in days	4 (2–6)	365
Wheeze within previous 12 months	80 (23.0%)	348
Fever	308 (85.6%)	360
Prior antibiotic treatment*	233 (63.0%)	370
Previous hospital admission	76 (21.1%)	360
Respiratory rate (breaths per minute)		
2–11 months	68 (60–77)	228
12–59 months	63 (55–72)	117
Wheeze	87 (23.6%)	369
Oxygen saturation <90%	174 (50.7%)	329
Inability to drink	126 (34.0%)	369
Central cyanosis	24 (6.5%)	367
Grunting	90 (24.4%)	369
Head nodding	108 (29.4%)	367
Altered consciousness	40 (10.8%)	369
Confirmed HIV infection†	36 (10.6%)	341
Very severe pneumonia	201 (54.3%)	370

* Patient-held documentation or history of specific, recent antibiotics received for the presenting episode of illness.

† >18 months *n* = 5; <18 months and PCR positive *n* = 31.

### Prevalence and correlates of TF

Our *a priori* definition of TF included a senior clinician's decision to change therapy, clinical indicators of deterioration (including death within 5 days of admission), absence of improvement after 48 h of therapy, or microbiological or radiological indications for switching treatment where this information was available (see Table 2). Based on these original criteria, 100/370 (27.0%; 95% CI 22.6–31.9) children failed treatment overall (21 [12.4% 95% CI 7.9–18.4] SP and 79 [39.3%; 95% CI 32.9–46.4] VSP), of whom 23 (6.2%; 95% CI 4.0–9.2) died (two SP and 21 VSP) (Table 4). The major reason for meeting this TF endpoint was, however, a decision by the ward clinician to change therapy without clinical indicators of TF (Table 5). Therefore, a *post-hoc* alternative definition of TF was applied, which categorised as treatment success children whose treatment was changed despite independent follow-up evaluations at 24 and 48 h indicating absence of clinical grounds for TF. Based on this new criterion, overall TF rates of 46/370 (12.4%; 95% CI 9.2–16.2) were observed with rates of 3/169 (1.8%; 95% CI 0.4–5.1) for SP and 43/201 (21.4%; 95% CI 15.9–27) for VSP (Table 4). Among children with SP who met the initial definition of TF, the most common change in therapy was prescription of additional gentamicin observed in 10/21 (47.6%) of cases. Exclusion of children categorised as TF in the absence of supportive clinical evidence from the alternative definition resulted in TF rates of 3/151 (2.0%; 95% CI 0.4–5.7) and 43/165 (26.1%; 95% CI 19.5–33.5) for SP and VSP, respectively.

**Table 4:** Prevalence of TF and mortality by pneumonia severity classification

Pneumonia severity classification	TF 1 – *a priori* definition Frequency (%, 95% CI)	TF 2 – Alternative definition Frequency (%, 95% CI)	*N*
All SP	21 (12.4, 7.9–18.4)	3 (1.8, 0.4–5.1)	169
SP/benzyl penicillin (group I)	17* (17.2, 10.3–26.1)	2 (2.0, 0.3–7.1)	99
SP/benzyl penicillin and gentamicin/other antibiotics (group II)	4 (5.7, 1.6–14.0)	1 (1.4, 0.04–7.7)	70
All VSP	79 (39.3, 32.5–46.4)	43 (21.4, 15.9–27.7)	201
VSP/benzyl penicillin and gentamicin (group III)	56† (41.8, 33.3–50.6)	28 (20.9, 14.4–28.8)	134
VSP/benzyl penicillin (group IV)	5‡ (15.2, 5.1–31.9)	1 (3.0, 0.1–15.8)	33
VSP/other antibiotics (group V)	18§ (52.9, 35.1–70.2)	14 (41.2, 24.6–59.3)	34
Total	100 (27.0, 22.6–31.9)	46 (12.4, 9.2–16.2)	370

SP, severe pneumonia; TF, treatment failure; VSP, very severe pneumonia.

* Two deaths.

† 14 deaths.

‡ One death.

§ Six deaths.

**Table 5:** Reasons for TF among children with severe forms of pneumonia

Reasons for TF	Frequency (%)
Clinician's decision to change treatment in absence of other criteria	54 (54.0)
Death	23 (23.0)
Persistence of tachypnoea, fever and indrawing	25 (25.0)
Documented signs of deteriorating clinical status	12 (12.0
Radiological findings informing change of treatment	2 (2.0)
Bacteriological findings informing change of treatment	1 (1.0)
Total	100

TF, treatment failure.

Total proportions exceed 100% due to overlap of criteria for TF for some patients.

The high frequency of alternative regimen use, with no clear clinical indication, in those with SP would mean attempts to examine associations with our *a priori* definition of TF essentially examine associations with use of non-recommended regimens. As employing our *post-hoc* definition yielded very few TF events and deaths in children with SP, these analyses were therefore not pursued.

Among children with VSP predictors of TF were more consistent using both pre-defined and *post-hoc* definitions in univariate analyses (Table 6). In a multivariable model with TF using our *a priori* definition as the outcome we included age, sex, Hib vaccination status, severe acute malnutrition as *a priori* covariates and all variables which were associated with TF in univariate analyses (inability to drink, altered consciousness, grunting, wheeze, antibiotic treatment given and HIV antibody status). Factors found to be independently associated with TF were grunting (OR 3.70, *P* = 0.001), younger age group – 2–11 months (OR 2.41, *P* = 0.05), inability to drink (OR 2.49, *P* = 0.02) and confirmed HIV-positive status (OR 3.05, *P* = 0.04). Characteristics associated with mortality were female gender (OR 10.25, *P* = 0.01), inability to drink (OR 9.72, *P* = 0.05) and rapid antibody test positive HIV status (OR 8.70, *P* = 0.03).

**Table 6:** Unadjusted odds ratios for predictors of TF and mortality among children with very severe pneumonia

Predictor	Risk of TF 1 – *a priori* definition OR (95% CI)	*P*	Risk of TF 2 – alternative definition OR (95% CI)	*P*	Risk of mortality OR (95% CI)	*P*
Age (2–11 *vs*. 12–59 months)	2.08 (1.05–4.10)	0.03	3.00 (1.17–7.74)	0.02	1.80 (0.57–5.67)	0.31
Female gender	1. 45 (0.81–2.59)	0.21	1.69 (0.83–3.42)	0.14	5.61 (1.55–20.39)	0.003
Hib* immunization status up to date	0.76 (0.43–1.34)	0.34	0.79 (0.41–1.49)	0.44	0.70 (0.26–1.89)	0.49
Severe malnutrition†	1.33 (0.74–2.39)	0.33	1.56 (0.78–3.13)	0.21	1.91 (0.67–4.92)	0.23
Central cyanosis	1.67 (0.71–3.97)	0.24	1.58 (0.61–4.11)	0.34	1.85 (0.56–6.08)	0.30
Inability to drink	2.08 (1.12–3.90)	0.02	2.27 (1.03–4.99)	0.04	6.39 (1.40–29.23)	0.006
Altered consciousness	2.58 (1.26–5.31)	0.008	2.09 (0.96–4.55)	0.06	3.58 (1.36–9.43)	0.006
Head nodding	0.92 (0.52–1.63)	0.77	0.79 (0.40–1.57)	0.51	1.02 (0.40–2.59)	0.97
Grunting	2.33 (1.29–4.23)	0.004	1.74 (0.88–3.46)	0.11	2.15 (0.84–5.50)	0.10
Hypoxia (SpO_2_ <90%)	0.94 (0.51–1.74)	0.95	0.83 (0.40–1.70)	0.61	0.71 (0.27–1.86)	0.49
Wheeze on admission	0.37 (0.17–0.79)	0.01	0.45 (0.17–1.15)	0.09	0.31 (0.07–1.42)	0.11
Previous hospital admission	0.63 (0.29–1.37)	0.24	0.86 (0.35–2.14)	0.75	1.05 (0.33–3.35)	0.93
Benzyl penicillin/gentamicin *vs*. benzyl penicillin alone	0.25 (0.09–0.68)	0.007	0.12 (0.02–0.90)	0.04	0.27 (0.03–2.11)	0.21
Benzyl penicillin/gentamicin *vs*. other antibiotics	1.84 (0.84–4.00)	0.13	2.82 (1.26–6.30)	0.01	1.84 (0.65–5.20)	0.25
HIV rapid antibody test positive	1.91 (0.77–4.72)	0.16	2.88 (1.08–7.68)	0.03	2.46 (0.72–8.46)	0.14

TF, treatment failure.

* *Haemophilus influenzae* type b.

† Visible severe wasting or oedema of both feet due to kwashiorkor.

We explored differences in baseline characteristics across the treatment subgroups of children within their severity strata. Continuous data plotted on histograms showed deviation from the normal distribution. We therefore used the Kruskal–Wallis test to compare medians and chi-square test for categorical data. Significant variation between subgroups would suggest that decisions to deviate from the recommended treatment guidelines were not random. In children with SP, we found those started on recommended treatment had shorter duration of illness (*P* = 0.04) and were more likely to have a history or signs of wheeze at initial assessment (OR 2.03, *P* = 0.02). Among those with VSP, children on recommended treatment had received antibiotics prior to admission less frequently (OR 2.76, *P* = 0.02) but were less likely to have altered consciousness (OR 3.23, *P* < 0.001) or inability to drink/breastfeed (OR 1.94, *P* = 0.02) than those given non-recommended treatments. A regimen of penicillin monotherapy, despite meeting the case definition for VSP, was associated with the absence of grunting (OR 2.14, *P* = 0.004).

The clinical outcomes of children with VSP treated in accordance to the guidelines (134/168) were compared with those who received alternative broad-spectrum antibiotics (mainly third-generation cephalosporins) (34/168). The crude risk difference of TF was 0.20 (95% CI 0.02–0.38);a final model comparing the treatment groups using propensity scores calculated to account for baseline imbalances between the groups yielded a risk difference of 0.37 (95% CI −0.84–0.51).

### Aetiological diagnoses

Samples were collected for blood culture on 338/385 (87.8%) children. Of this group, 54 (16%) cultures were positive but 43 (13%) samples yielded contaminants (including 29 isolates of coagulase negative Staphylococci); a clinically significant organism was isolated from 11 (3.3%) of blood cultures (five *Streptococcus pneumoniae*, three *Salmonella typhimurium*, two *Escherichia coli* and one *Pseudomonas aeruginosa*). The child from whom *Pseudomonas* was isolated died.

We screened 342/385 (89%) children for HIV of whom 38 (11.1%) were positive on rapid antibody test – five aged above 18 months and 33 below 18 months (median age 5.4 months). Confirmatory PCR testing for HIV diagnosis was done in 32/33 rapid antibody positive children aged below 18 months (one patient died before a PCR was obtained). HIV prevalence in the study population was 10.6% (37/341) and varied between subgroups – 8.2% (13/159) in children with SP and 12.6% (23/182) in those with VSP.

## Discussion

We conducted a comprehensive audit at KNH to determine the clinical effectiveness of WHO case management guidelines for children admitted with community-acquired SP or VSP. The population studied was drawn from the Kenyan National Hospital, and among admitted children, 46% had SP and 54% VSP. This contrasts with a recent report from a Kenyan district hospital reporting a ratio of 3:1 for SP:VSP (Nokes *et al.* 2009), but is more consistent with findings from a tertiary hospital in South Africa with a high HIV prevalence, where 71% of children hospitalised with pneumonia had VSP (McNally *et al.* 2007). Higher proportions of sicker children in tertiary facilities may suggest that they do not provide a representative picture of pneumonia epidemiology; unfortunately, there are very few data available to address this question. Of children enrolled, 21% were reported to have had at least one previous hospital admission and 64% reported treatment with antibiotics prior to admission. Although the reliability of caretakers’ reports has been challenged (Hildenwall *et al.* 2009), these factors, in addition to the short period over which data were collected and the resultant potential for seasonal bias, must also be considered when interpreting our findings. Despite these limitations, we consider that this study provides useful, new data to inform discussions on the importance of TF in SP and VSP from a Kenyan perspective.

### TF in children with SP

The definition for TF in childhood acute respiratory infections remains a topic of discussion (Hazir *et al.* 2006; Ayieko & English 2007). Using our *a priori* definition, and after excluding reversible airways disease as a major cause of initially severe illness, we observed a TF rate of 12.4%; a figure lower than the 19% reported from a large multicentre trial recruiting predominantly Asian children (Addo-Yobo *et al.* 2004) but comparable to a rate of 12% reported in a prospective cohort study conducted in a district hospital in coastal Kenya (Webb *et al.* 2012). The conditions under which our study was conducted were not tightly controlled, and change of antibiotics, one of the criteria for TF, was often done within 24 h of admission and appeared frequently to lack an obvious clinical, radiological or laboratory basis. We therefore used an alternative definition of TF, omitting the change of antibiotics not accompanied by clinical or other indications for change. This new definition revealed a far lower TF rate of 1.8% children for children with SP against a mortality of 1.2%. This mortality rate is similar to that recently published for children with a similar classification of 0.2% and 0.7% (Addo-Yobo *et al.* 2004). However, 36% of this group received broader spectrum antibiotics than recommended from early in the course of their admission, a practice which was more likely for a child with longer symptom history and less likely if they were wheezing but not associated with prior antibiotic use, presence of hypoxaemia or if they were HIV infected.

### TF in children with VSP

Both TF and mortality were considerably higher in children classified as having VSP, compared to those with SP TF. Our *a priori* definition of 39.3% (21.4% by our alternative definition) compares with the 13.6% TF rate (mortality 6.6%) reported in a multi-country trial of 958 predominantly Asian children in which recruitment at the Zambian site was stopped owing to high mortality (Asghar *et al.* 2008). This high TF rate includes 21 deaths (10.5%). Consistent with previous studies, female gender (Spooner *et al.* 1989; Asghar *et al.* 2008), inability to drink (Shann *et al.* 1989) and a positive HIV status (Jeena *et al.* 2006) were associated with increased risk of mortality in multivariable analysis of children with VSP. The high TF rate, even with our modified definition, could indicate suboptimal effectiveness of the current empiric treatment regimen in our setting. Alternatively, it is possible that the severity of disease at presentation and absence of advanced supportive care contribute to high TF and mortality. Only appropriate and large randomised and controlled trials of alternative antibiotic regimens or other interventions are likely to resolve uncertainties and provide definitive evidence to inform future guidelines.

Other findings of note include the inconsistency in adherence to guidelines that was associated with possible under-treatment (though with good outcomes) in some children meeting WHO criteria for VSP and possible over-treatment in some (with longer duration of illness and lower prevalence of wheeze) meeting criteria for SP. Difficulty in ensuring compliance with guidelines is well-recognised (English *et al.* 2004), and it is our observation that clinicians often feel monotherapy with penicillin is inadequate for children admitted with SP. Unfortunately an almost complete absence of data on treatment effectiveness means such beliefs have been hard to refute or support. Our data would suggest penicillin monotherapy (Addo-Yobo *et al.* 2004, 2011; Hazir *et al.* 2008) might remain adequate treatment for HIV-negative Kenyan children who are appropriately classified as having SP. Indeed, evidence from trials conducted in predominantly Asian populations have shown oral amoxicillin to be an effective alternative to benzyl penicillin for SP. Generating similar evidence for sub-Saharan populations would however, require large non-inferiority or equivalence trials with strict definitions of TF. Unfortunately the capacity for conducting such trials is extremely limited. While there have been major investments in the ability to conduct trials among African children with malaria, tuberculosis and HIV, the same is not true for pneumonia, the top cause of child mortality globally (Liu *et al.* 2012).

Our study confirmed a high prevalence of HIV among children with pneumonia supporting the routine provider-initiated counselling and testing strategies for HIV in admitted children and the use of rapid tests to guide the immediate management, including treatment for pneumocystis, rather than awaiting PCR confirmation.

In post-hoc analyses of the data from children with VSP, we used propensity scores to correct baseline imbalances between children treated with the guideline-recommended antibiotics and those who received more expensive, broader spectrum treatments and found no difference in TF rates between the two groups. This finding challenges the common belief by clinicians that broader spectrum antibiotic treatments are more effective than guideline-recommended empiric treatments. It is however important to note that this study was neither designed, nor powered, to compare antibiotic regimens. The results of this comparative analysis should therefore be interpreted with caution.

All analyses were conducted within the separate populations of children initially classified as having SP or VSP and results should be interpreted with caution as no allowance was made for multiple hypothesis testing. Fewer children presented with SP than we anticipated and the prevalence of TF within this group was low. These factors led us to abandon the objective to determine risk factors for TF in this subgroup. Among children with VSP, the number recruited (214) provided power to detect odds ratios >2 for risk factors with a prevalence of 20%. Whereas there was an effort to screen all children for eligibility, 106 children (18% of all possible cases) were admitted with pneumonia and not studied. Demographical and outcome data for these children collected retrospectively were found to be comparable to those of the patients enrolled (data not shown). The failure to recruit all potentially eligible children was commonly because recruiting investigators (paediatric trainees) had continuing commitments to routine care and there were inadequate funds for additional dedicated research staff.

## Conclusion

While TF is frequently used as a measure of outcome in clinical studies, we found that the rate differs greatly depending on the definition used. This was particularly so in children with SP in whom there was a tendency to revise management in the absence of any apparent supportive clinical, laboratory or radiological evidence. There is a clear difference between populations of children with carefully classified SP and VSP, with relatively low rates of TF and mortality in children with the former. These findings offer some support to current recommendations for monotherapy in the treatment of SP but further data, with better enforcement of guideline-directed therapy are required. Further, our study revealed no difference in clinical outcomes among children treated with standard treatments and those perceived to be more effective; however, the appropriate studies to address clinical questions of this nature would be locally conducted randomized controlled trials which are currently poorly supported. Building capacity for measuring treatment effectiveness and the conduct of trials is urgently needed if global targets for reduction in childhood pneumonia mortality are to be met.

## References

[b1] Addo-Yobo E, Chisaka N, Hassan M (2004). Oral amoxicillin versus injectable penicillin for severe pneumonia in children aged 3 to 59 months: a randomised multicentre equivalency study. The Lancet.

[b2] Addo-Yobo E, Anh DD, El-Sayed HF (2011). Outpatient treatment of children with severe pneumonia with oral amoxicillin in four countries: the MASS study. Tropical Medicine and International Health.

[b3] Adegbola RA, Secka O, Lahai G (2005). Elimination of *Haemophilus influenzae* type b (Hib) disease from The Gambia after the introduction of routine immunisation with a Hib conjugate vaccine: a prospective study. The Lancet.

[b4] Agweyu A, Opiyo N, English M (2012). Experience developing national evidence based clinical guidelines for childhood pneumonia in a low-income setting – making the GRADE?. BMC Pediatrics.

[b5] Asghar R, Banajeh S, Egas J (2008). Chloramphenicol versus ampicillin plus gentamicin for community acquired very severe pneumonia among children aged 2-59 months in low resource settings: multicentre randomised controlled trial (SPEAR study). BMJ.

[b6] Ayieko P, English M (2007). Case management of childhood pneumonia in developing countries. The Pediatric Infectious Disease Journal.

[b7] Berkley JA, Maitland K, Mwangi I (2005). Use of clinical syndromes to target antibiotic prescribing in seriously ill children in malaria endemic area: observational study. BMJ.

[b8] Cowgill KD, Ndiritu M, Nyiro J (2006). Effectiveness of *Haemophilus influenzae* type b Conjugate vaccine introduction into routine childhood immunization in Kenya. JAMA.

[b9] English M, Esamai F, Wasunna A (2004). Assessment of inpatient paediatric care in first referral level hospitals in 13 districts in Kenya. The Lancet.

[b12] Graham SM (2003). HIV and respiratory infections in children. Current Opinion in Pulmonary Medicine.

[b13] Guyatt GH, Oxman AD, Vist GE (2008). GRADE: an emerging consensus on rating quality of evidence and strength of recommendations. BMJ.

[b14] Hazir T, Qazi SA, Nisar YB (2006). Can WHO therapy failure criteria for non-severe pneumonia be improved in children aged 2-59 months?. The International Journal of Tuberculosis and Lung Disease.

[b15] Hazir T, Fox LM, Nisar YB (2008). Ambulatory short-course high-dose oral amoxicillin for treatment of severe pneumonia in children: a randomised equivalency trial. The Lancet.

[b16] Hildenwall H, Lindkvist J, Tumwine JK (2009). Low validity of caretakers’ reports on use of selected antimalarials and antibiotics in children with severe pneumonia at an urban hospital in Uganda. Transactions of the Royal Society of Tropical Medicine and Hygiene.

[b17] Irimu G, Wamae A, Wasunna A (2008). Developing and introducing evidence based clinical practice guidelines for serious illness in Kenya. Archives of Disease in Childhood.

[b18] Irimu GW, Gathara D, Zurovac D (2012). Performance of health workers in the management of seriously sick children at a kenyan tertiary hospital: before and after a training intervention. PLoS One.

[b19] Jeena P, Thea DM, MacLeod WB (2006). Failure of standard antimicrobial therapy in children aged 3-59 months with mild or asymptomatic HIV infection and severe pneumonia. Bulletin of the World Health Organization.

[b100] Kenya National Bureau of Statistics (KNBS) and ICF Macro (2010). Kenya Demographic Health Survey 2008–09.

[b20] Kourtis AP, Bansil P, Posner SF, Johnson C, Jamieson DJ (2007). Trends in hospitalizations of HIV-infected children and adolescents in the United States: analysis of data from the 1994–2003 Nationwide Inpatient Sample. Pediatrics.

[b21] Liu L, Johnson HL, Cousens S (2012). Global, regional, and national causes of child mortality: an updated systematic analysis for 2010 with time trends since 2000. The Lancet.

[b22] Madhi SA, Petersen K, Madhi A, Wasas A, Klugman KP (2000). Impact of human immunodeficiency virus type 1 on the disease spectrum of *Streptococcus pneumoniae* in South African children. The Pediatric Infectious Disease Journal.

[b23] McNally LM, Jeena PM, Gajee K (2007). Effect of age, polymicrobial disease, and maternal HIV status on treatment response and cause of severe pneumonia in South African children: a prospective descriptive study. The Lancet.

[b200] Ministry of Health (Government of Kenya) (2006). Guidelines for HIV Testing in Clinical Settings.

[b24] Mulholland K, Hilton S, Adegbola R (1997). Randomised trial of *Haemophilus influenzae* type-b tetanus protein conjugate vaccine [corrected] for prevention of pneumonia and meningitis in Gambian infants. The Lancet.

[b25] Murphy TV, Pastor P, Medley F, Osterholm MT, Granoff DM (1993). Decreased Haemophilus colonization in children vaccinated with *Haemophilus influenzae* type b conjugate vaccine. Journal of Pediatrics.

[b26] Niessen LW, ten Hove A, Hilderink H, Weber M, Mulholland K, Ezzati M (2009). Comparative impact assessment of child pneumonia interventions. Bulletin of the World Health Organization.

[b27] Nokes DJ, Ngama M, Bett A (2009). Incidence and severity of respiratory syncytial virus pneumonia in rural Kenyan children identified through hospital surveillance. Clinical Infectious Diseases.

[b28] Nyandiko WM, Greenberg D, Shany E, Yiannoutsos CT, Musick B, Mwangi AW (2007). Nasopharyngeal *Streptococcus pneumoniae* among under-five year old children at the Moi Teaching and Referral Hospital, Eldoret, Kenya. East African Medical Journal.

[b29] Oniyangi O, Awani B, Iregbu KC (2006). The pattern of paediatric HIV/AIDS as seen at the National Hospital Abuja Nigeria. Nigerian Journal of Clinical Practice.

[b30] Sazawal S, Black R (2003). Pneumonia Case Management Trials Group. Effect of pneumonia case management on mortality in neonates, infants, and preschool children: a meta-analysis of community-based trials. The Lancet.

[b31] Scott JA, English M (2008). What are the implications for childhood pneumonia of successfully introducing Hib and pneumococcal vaccines in developing countries?. PLoS Medicine.

[b32] Scott JA, Hall AJ, Hannington A (1998). Serotype distribution and prevalence of resistance to benzylpenicillin in three representative populations of *Streptococcus pneumoniae* isolates from the coast of Kenya. Clinical Infectious Diseases.

[b33] Shann F, Barker J, Poore P (1989). Clinical signs that predict death in children with severe pneumonia. The Pediatric Infectious Disease Journal.

[b34] Spooner V, Barker J, Tulloch S (1989). Clinical signs and risk factors associated with pneumonia in children admitted to Goroka Hospital, Papua New Guinea. Journal of Tropical Pediatrics.

[b35] Vella V, Tomkins A, Ndiku J, Marshall T (1992). Determinants of child mortality in south-west Uganda. Journal of Biosocial Science.

[b36] Watt JP, Wolfson LJ, O'Brien KL (2009). Burden of disease caused by *Haemophilus influenzae* type b in children younger than 5 years: global estimates. The Lancet.

[b37] Webb C, Ngama M, Ngatia A (2012). Treatment failure among Kenyan children with severe pneumonia – a cohort study. The Pediatric Infectious Disease Journal.

[b38] Wenger JD (1998). Epidemiology of *Haemophilus influenzae* type b disease and impact of *Haemophilus influenzae* type b conjugate vaccines in the United States and Canada. The Pediatric Infectious Disease Journal.

[b39] WHO (1990). Acute Respiratory Infections in Children: Case Management in Small Hospitals in Developing Countries.

[b40] Zar HJ, Apolles P, Argent A (2001). The etiology and outcome of pneumonia in human immunodeficiency virus-infected children admitted to intensive care in a developing country. Pediatric Critical Care Medicine.

[b41] Zwi KJ, Pettifor JM, Soderlund N (1999). Paediatric hospital admissions at a South African urban regional hospital: the impact of HIV, 1992–1997. Annals of Tropical Paediatrics.

